# Analysis of GAGE, NY-ESO-1 and SP17 cancer/testis antigen expression in early stage non-small cell lung carcinoma

**DOI:** 10.1186/1471-2407-13-466

**Published:** 2013-10-08

**Authors:** Morten F Gjerstorff, Mette Pøhl, Karen E Olsen, Henrik J Ditzel

**Affiliations:** 1 Department of Cancer and Inflammation Research, Institute for Molecular Medicine (IMM), University of Southern Denmark, Winsloewparken 25, 3, Odense C, DK-5000, Denmark; 2 Institute of Clinical Research, University of Southern Denmark, Campusvej 55, Odense, DK-5230, Denmark; 3 Department of Oncology, Odense University Hospital, Sdr. Boulevard 29, Odense, DK-5000, Denmark; 4 Department of Pathology, Odense University Hospital, Winsloewparken 15, Odense, DK-5000, Denmark

**Keywords:** Cancer/testis antigen, Immunotherapy, GAGE, NY-ESO-1, SP17, Lung cancer

## Abstract

**Background:**

The unique expression pattern and immunogenic properties of cancer/testis antigens make them ideal targets for immunotherapy of cancer. The MAGE-A3 cancer/testis antigen is frequently expressed in non-small cell lung cancer (NSCLC) and vaccination with MAGE-A3 in patients with MAGE-A3-positive NSCLC has shown promising results. However, little is known about the expression of other cancer/testis antigens in NSCLC. In the present study the expression of cancer/testis antigens GAGE, NY-ESO-1 and SP17 was investigated in patients with completely resected, early stage, primary NSCLC.

**Methods:**

Tumor biopsies from normal lung tissue and from a large cohort (n = 169) of NSCLC patients were examined for GAGE, NY-ESO-1 and SP17 protein expression by immunohistochemical analysis. The expression of these antigens was further matched to clinical and pathological features using univariate cox regression analysis.

**Results:**

GAGE and NY-ESO-1 cancer/testis antigens were not expressed in normal lung tissue, while SP17 was expressed in ciliated lung epithelia. The frequency of GAGE, NY-ESO-1 and SP17 expression in NSCLC tumors were 26.0% (44/169), 11.8% (20/169) and 4.7% (8/169), respectively, and 33.1% (56/169) of the tumors expressed at least one of these antigens. In general, the expression of GAGE, NY-ESO-1 and SP17 was not significantly associated with a specific histotype (adenocarcinoma vs. squamous cell carcinoma), but high-level GAGE expression (>50%) was more frequent in squamous cell carcinoma (p = 0.02). Furthermore, the frequency of GAGE expression was demonstrated to be significantly higher in stage II-IIIa than stage I NSCLC (17.0% vs. 35.8%; p = 0.02). Analysis of the relation between tumor expression of GAGE and NY-ESO-1 and survival endpoints revealed no significant associations.

**Conclusion:**

Our study demonstrates that GAGE, NY-ESO-1 and SP17 cancer/testis antigens are candidate targets for immunotherapy of NSCLC and further suggest that multi-antigen vaccines may be beneficial.

## Background

Harnessing the power of our immune system has long been a promising approach to treating cancer, and a large number of tumor antigens that might be used as targets for immunotherapy have been identified. Cancer/testis (CT) antigens are among the most promising due to their highly restricted expression to immune privileged cells of the testis and placenta in normal tissues as well as their natural immunogenic properties [1,2]. Current strategies employing CT antigens as targets for immunotherapy include vaccination [1,3] and adoptive transfer of T cells with genetically-modified T cell receptors [4-6].

Non-small cell lung cancer (NSCLC) accounts for more than 85% of new cases of lung cancer, which is the leading cause of cancer deaths worldwide [7], highlighting the need for novel therapeutic strategies for treatment of this disease. Cellular and humoral immune responses to CT antigens have been reported in NSCLC patients [8-14], suggesting that these proteins may be candidate targets for cancer immunotherapy of NSCLC. In addition, CT antigen sero-reactivity may be of diagnostic value for NSCLC patients [15]. Interestingly, adjuvant therapy with a MAGE-A3 CT antigen vaccine in patients with MAGE-A3-positive NSCLC has shown promising results [16], and allogeneic lymphocytes expressing recombinant T-cell receptors recognizing CT antigens NY-ESO-1 and MAGE-A3 were recently shown to effectively kill lung cancer cells [4]. This suggests that cancer immunotherapy targeting CT antigens may be an effective treatment for NSCLC. However, characterization of additional targets in NSCLC is needed to further develop broadly applicable, effective and specific immunotherapy regimens.

An important factor to consider when selecting appropriate targets for cancer immunotherapy is the expression frequency within the cancer of interest. In this study, we report a systematic analysis of the expression of the CT antigens GAGE, NY-ESO-1 and SP17 in early-stage NSCLC. NY-ESO-1 and the GAGE multi-gene family are members of the chromosome X-encoded CT antigens, which generally exhibit complete testis-specificity and are expressed at the spermatogonial stage of spermatogenesis [2]. In contrast, autosomal encoded CT antigens, such as SP17, are characterized by low expression in a limited number of non-testis, normal, tissues and tend to be expressed in the late stages of spermatogenesis. Our results will enhance the selection of appropriate targets for immunotherapeutic treatment of this disease.

## Methods

### Tumor samples

NSCLC surgical resection specimens were collected as diagnostic specimens from patients treated at the University Hospital of Odense from 1992–1999. The experiment was conducted in compliance with the Helsinki declaration and was approved by the ethical committee of Funen and Vejle County (VF20050069). Informed consent from participants was not needed for this type of experiment. All patients had undergone complete surgical resection without further treatment (neoadjuvant or adjuvant chemo- or radiotherapy). The histological subtypes of the tumors were established by morphology using light microscopy or by TTF1 and p63 status using immunohistochemistry. Formalin-fixed and paraffin-embedded tumor sections were stained with hematoxylin and eosin, and two 1 mm cores were punched from the central part of the tumors were transferred to tissue microarrays for further analysis.

### Immunohistochemical staining

Methods for immunohistochemical staining of GAGE, NY-ESO-1 and SP17 in formalin-fixed, paraffin-embedded tissues and the characteristics of the antibodies used have been described previously [17-19]. Notably, the anti-GAGE antibody likely recognizes all members of the GAGE family. In brief, tissue sections were cut, deparaffinized, treated with 1.5% H_2_0_2_ in Tris-buffered saline (pH 7.5) for 10 min to block endogenous peroxidase activity, rinsed in distilled H_2_O, demasked for antigen retrieval and washed in TNT buffer (0.1 M Tris, 0.15 M NaCl, 0.05% Tween-20, pH 7.5). Primary monoclonal antibodies (anti-GAGE (clone M3) [18], 1:100; anti-NY-ESO-1 (clone E978) [18] 1:25; anti-SP17 (clone 22) [17] 1:400) were diluted in antibody diluent (DAKO Cytomation, Glostrup, Denmark) and added to sections for 1 h at room temperature. Sections were washed with TNT and incubated with horseradish peroxidase-conjugated Envision or Powervision polymer (DAKO Cytomation) for 30 min, followed by another wash with TNT. The final reaction product was visualized by incubating with 3,3’-diaminobenzidine (DAB) + substrate-chromogen for 10 min, followed by washing with H_2_O and counterstaining of sections with Mayers hematoxylin before mounting in AquaTex (Merck Inc., Whitehouse Station, NJ, USA).

### Histological evaluation

Immunohistochemical staining was evaluated for percentage of positive tumor cells by a skilled pathologist. Since positively-stained cells were generally strongly stained, differences in intensity was not assessed. The specimens were scored in four categories: 0 (≤ 1%), 1 (>1% - ≤ 10%), 2 (>10% - ≤ 50%) and 3 (>50%). Cells were considered positive if staining was convincingly observed in either the cytoplasm or the nuclei, or both, regardless of intensity. The cores were reported as missing if none or few tumor cells were present (<30 cells).

### Statistical analysis

Univariate regression analysis using Cox proportional-hazard models and Kaplan-Meier survival analysis was performed using STATA software. The comparison of CT antigen expression with histotype and clinical stage was analyzed with the two-sided *chi-squared* test using a 5% significance level. Analysis of CT antigen co-expression was done with the Z test comparing expected and observed proportions of positive tumors.

## Results and discussion

**Figure 1:**
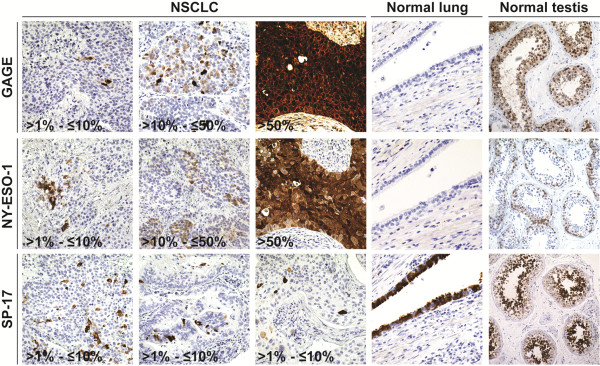
**Immunohistochemical analysis of GAGE, NY-ESO-1 and SP17 expression in clinical NSCLC specimens.** Representative tumor staining with monoclonal antibodies recognizing GAGE, NY-ESO-1 and SP17. Percentages of positive cells within tumors are indicated. Normal lung and testis were included as controls. Magnification: ×20 (lung and testis) and ×40 (tumors).

**Figure 2:**
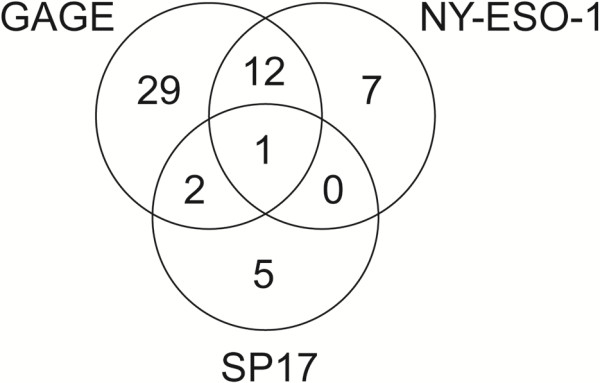
**Venn diagram demonstrating overlap between GAGE, NY-ESO-1 and SP17 expression in NSCLC specimens.** Numbers represent positive tumors among the total of 169 NSCLC specimens included in the analysis.

We evaluated the expression of GAGE, NY-ESO-1 and SP17 CT antigens in normal lung tissue (n = 5) and tumors from 169 patients with completely resected, early stage primary NSCLC. Patient characteristics are presented in Table 1. GAGE, NY-ESO-1 and SP17 expression was examined using well-characterized antibodies and previously established methods for immunohistochemical staining [17,18]. GAGE and NY-ESO-1 was not detected in normal lung tissues, but SP17 was expressed in a subset of ciliated epithelial cells of the bronchi (Figure 1), in accordance with previously published data [17,18]. As shown in Table 2, GAGE proteins were detected in 26.0% (44/169) of NSCLC tumors and in 63.6% (28/44) of the positive tumors there were more than 50% positive tumor cells. This demonstrates that the expression frequency of GAGE proteins in NSCLC is similar to that of MAGE-A3, which is currently being tested as a vaccine target in NSCLC [16,20], as mentioned above. All GAGE-positive tumor cells exhibited cytoplasmic staining, but there were clear differences in the level of nuclear staining among and within tumors, ranging from absent to intense (Figure 1). This subcellular distribution is in agreement with a previous report on GAGE protein expression in a small set of lung cancers and other types of cancer [18]. NY-ESO-1 was detected in 11.8% (20/169) of tumors and, as with GAGE, the distribution in most tumors was near homogenous (>50% positive tumor cells in 14 of 20 tumors examined) (Table 2). This expression frequency is in line with previous studies reporting 8.3-25% NY-ESO-1-positive NSCLC tumors [20-23]. The discrepancy in reported frequencies of NY-ESO-1 expression may be due to multiple parameters such as variation in clinical material (sampling, stage, subtype, treatment), staining protocol, scoring system/personnel etc. It is possible that the present study, using two 1 mm cores per tumor, may have included more false negatives than studies using whole tumor sections. However, NY-ESO-1 was relatively homogenously expressed in the majority of NSCLC tumors analyzed, supporting the validity of the two-core approach. The subcellular localization of NY-ESO-1 in NSCLC tumors was predominantly cytoplasmic. SP17 was detected in 4.7% (8/169) NSCLC tumors, which is comparable to existing targets of NSCLC (e.g. EML4-ALK) [24,25]. In all 8 positive tumors, less than 10% of the tumor cells were positive. Notably, the SP17-positive tumor cells exhibited a scattered distribution within tumors in contrast to GAGE and NY-ESO-1, which were most often either homogenously expressed or clustered. CT antigens have been proposed as markers of cancer stem cells [26], and further studies should be conducted to uncover the identity of this small subset of SP17-positive tumor cells. It is also notable that while the frequency of tumors positive for both GAGE and NY-ESO-1 proteins suggested a degree of coordinated expression of these proteins (p = 0.03), neither showed any tendency to co-expression with SP17 (p = 0.65 and p = 1.00) (Figure 2). Unlike GAGE and NY-ESO-1, SP17 is expressed in ciliated normal tissues in addition to testis, indicating that the encoding genes exhibit differences in tissue-specific regulation, which may explain the significant expression dissimilarities observed in NSCLC and other cancers [17]. It further confirms the notion that chromosome X encoded and autosomal encoded CT antigens exhibit different expression profiles in normal and malignant tissues.

**Table 1:** Characteristics of patients included in the study

**Pathological parameter**		**Cases**	**%**
**Gender**	Male	104	62.1
	Female	65	37.9
**Histology**	Adenocarcinoma	81	47.9
	Squamous cell carcinoma	84	49.7
	NOS*	4	2.4
**TNM stage**	IA	31	18.3
	IB	57	33.7
	IIA	40	23.7
	IIB	17	10.1
	IIIA	24	14.2

*NOS 'Not otherwise specified’. Negative for adenocarcinoma marker TFF1 and squamous cell carcinoma marker p63.

**Table 2:** Expression of GAGE, NY-ESO-1 and SP17 in human NSCLC

**Tumor expression of antigen**	**Histotype**			**All NSCLC specimens (n = 169)**
	**Adenocarcinoma (n = 81)**	Squamous cell carcinoma (n = 88)^*^	**p-value**	
GAGE	15 (18.5%)	29 (33.0%)	0.19	44 (26.0%)
>1% - ≤ 10%	5 (6.2%)	5 (5.7%)		
>10% - ≤ 50%	2 (2.5%)	4 (4.6%)		
>50%	8 (9.9%)	20 (22.7%)	0.02	
NY-ESO-1	8 (9.9%)	12 (13.6%)	0.49	20 (11.8%)
>1% - ≤ 10%	2 (2,5%)	1 (1.1%)		
>10% - ≤ 50%	2 (2.5%)	1 (1.1%)		
>50%	4 (4.9%)	10 (11.4%)	0.13	
SP17	4 (4.9%)	4 (4.6%)	0.76	8 (4.7%)
>1% - ≤ 10%	4 (4.9%)	4 (4.6%)		
>10% - ≤ 50%	0	0		
>50%	0	0		

^*^ The survival of the four NOS patients resembled that of patients with squamous cell carcinoma and were therefore included in this group for statistical analysis.

The panel of NSCLC included both adenocarcinomas and squamous cell carcinomas. In general, the expression of GAGE, NY-ESO-1 and SP17 CT antigens were not associated with any specific histology type (Table 2), but strongly GAGE-positive tumors (>50% positive tumor cells) were more frequent in squamous cell carcinomas (p = 0.02). A similar correlation has been reported between MAGE-A3 and MAGE-A4 and squamous cell carcinoma [20]. Adenocarcinomas and squamous cell carcinomas have been shown to differ in their DNA methylation patterns [27,28], and since promoter hypomethylation is important inducer of CT antigen gene expression in cancer cells [2], this may explain the differences in CT antigen expression between these two subtypes of NSCLC.

GAGE protein expression significantly correlated with disease progression (p = 0.02), i.e. 17.0% (15/88) of stage I and 35.8% (29/81) of stage II-IIIa tumors were GAGE-positive (Table 3). NY-ESO-1 expression also tended to associate with advanced disease stages, but not to a statistically significant degree. Similarly, the frequency of MAGE-A4-positive tumors has been reported to be a significantly higher in stage II-IV than stage I NSCLC [20].

**Table 3:** Expression of GAGE, NY-ESO-1 and SP17 in different stages of NSCLC

**Antigen**	**TNM stage**		
	**Ia-b (n = 88)**	**II-IIIa (n = 81)**	**p-value**
GAGE	15 (17.0%)	29 (35.8%)	0.02
>1% - ≤ 10%	4 (4.5%)	6 (7.4%)	
>10% - ≤ 50%	0	6 (7.4%)	
>50%	11 (12.5%)	17 (21.0%)	
NY-ESO-1	9 (10.2%)	11 (13.6%)	0.36
>1% - ≤ 10%	0	3 (3.7%)	
>10% - ≤ 50%	2 (2.3%)	1 (1.2%)	
>50%	7 (7.9%)	7 (8.7%)	
SP17	4 (4.5%)	4 (4.9%)	0.94
>1% - ≤ 10%	4 (4.5%)	4 (4.9%)	
>10% - ≤ 50%	0	0	
>50%	0	0	

**Figure 3:**
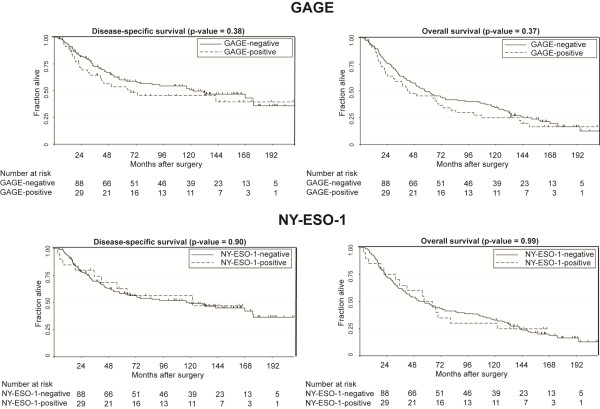
**Kaplan-Meier plots evaluating disease-specific and overall survival of NSCLC patients, according to GAGE and NY-ESO-1 expression, are shown.** Log-rank test was used.

The association between CT antigen expression and disease-specific and overall survival was also analyzed for GAGE and NY-ESO-1 (Figure 3); SP-17-positive specimen numbers were too low to allow for a statistical analysis. Although GAGE expression tended to correlate with poor survival, neither GAGE nor NY-ESO-1 was significantly associated with disease-specific or overall survival.

Our results show that the CT antigens GAGE, NY-ESO-1 and SP17 are expressed in a considerable proportion of NSCLC and may therefore serve as candidate targets for immunotherapeutic treatments of this disease. Furthermore, GAGE and NY-ESO-1 were present in more than 50% of the tumor cells in 63.6% (28/44) and 70% (14/20) of the positive cases, respectively. It seems likely that treatment directed against a tumor antigen broadly expressed within tumors may be most effective, although this has not been demonstrated. The relative homogeneity of GAGE and NY-ESO-1 in NSCLC tumors further strengthens their therapeutic potential, while the scattered expression of SP17 in NSCLC tumors suggests that this is a relatively poor target for NSCLC. Our results demonstrate significant differences in tumor expression of the two chromosome X-encoded CT antigens GAGE, NY-ESO-1 and the autosomal CT antigen SP17 in NSCLC. While only one tumor was positive for all three CT antigens, 56/169 (33.1%) were positive for at least one of these CT antigens, demonstrating that immunotherapeutic strategies should aim at different CT antigen targets, including both chromosome X-encoded and autosomal encoded antigens.

## Conclusions

This study determines the expression frequency and correlation with clinical parameters of GAGE, NY-ESO-1 and SP17 CT antigens in NSCLC, which may facilitate the use of these CT antigens as therapeutic targets for immunotherapy of NSCLC.

## Abbreviations

CT: Cancer/testis antigen; NSCLC: Non-small cell lung carcinoma.

## Competing interests

The authors declare that they have no competing interests.

## Authors’ contributions

MG designed the experiments, analyzed the data and prepared the manuscript. MP designed the experiments, analyzed the data and prepared the manuscript. KEO designed the experiments and analyzed the data. HJD designed the experiments and prepared the manuscript. All authors read and approved the final manuscript.

## Pre-publication history

The pre-publication history for this paper can be accessed here:

http://www.biomedcentral.com/1471-2407/13/466/prepub
